# AX-2: A Promising Non-Hemolytic Protein of *Bacillus thuringiensis* with Potent Selective Cytotoxicity Against Breast Cancer Cells

**DOI:** 10.3390/molecules31030475

**Published:** 2026-01-29

**Authors:** Alain Cruz-Nolasco, Miguel Angel Peña-Rico, Sibel J. Estrada-Escobedo, Angel A. Ortela-Gregorio, Erick A. Juarez-Arellano, Genaro Vázquez-Victorio, Angelica S. Martinez-Ramirez, Michele Rorato Sagrillo, Roberto C. Vianna Santos, Luz Camacho, Nayeli G. Nieto-Velázquez, A. Karin Navarro-Mtz

**Affiliations:** 1 División de Estudios de Posgrado, Doctorado en Biotecnología, Universidad del Papaloapan, Circuito Central 200, Parque Industrial, Tuxtepec 68301, Oaxaca, Mexico; alandurst13@gmail.com; 2 Centro de Investigaciones Científicas, Instituto de Biotecnología, Universidad del Papaloapan, Circuito Central 200, Parque Industrial, Tuxtepec 68301, Oaxaca, Mexico; asmartinez@unpa.edu.mx; 3 División de Estudios de Posgrado, Maestría en Biotecnología, Universidad del Papaloapan, Circuito Central 200, Parque Industrial, Tuxtepec 68301, Oaxaca, Mexico; 4 Centro de Investigaciones Científicas, Instituto de Química Aplicada, Universidad del Papaloapan, Circuito Central 200, Parque Industrial, Tuxtepec 68301, Oaxaca, Mexico; eajuarez@unpa.edu.mx; 5 Facultad de Ciencias, Universidad Nacional Autónoma de México, Circuito Exterior S/N, Ciudad Universitaria, Ciudad de Mexico 04510, Mexico; 6 Graduate Program in Nanoscience, Franciscan University, Santa Maria 97010-032, RS, Brazil; 7 Oral Microbiology Laboratory, Universidade Federal de Santa Maria, Santa Maria 97065-060, RS, Brazil; 8 Laboratorio de Nutrición Experimental, Instituto Nacional de Pediatría, Ciudad de Mexico 04530, Mexico; 9 División de Investigación, Hospital Juárez de México, Ciudad de Mexico 07760, Mexico

**Keywords:** parasporin ≈ 55 kDa, apoptosis, MCF-7, anticancer, oxidative stress

## Abstract

Conventional cancer therapies often harm healthy tissues due to their poor specificity, resulting in significant side effects that diminish patients’ quality of life. Parasporins, a group of non-hemolytic parasporal proteins produced by *Bacillus thuringiensis*, are known for their selective cytotoxicity toward cancer cells. Typically, these proteins require activation through physical or biochemical treatments that fragment them into multiple peptides of varying sizes, which are then tested as mixtures, without purification, against cancer cell lines. In this study, a purification strategy that isolates the protein without prior activation and evaluates the resulting cytotoxic mechanism is proposed. The purification consists of four steps: (1) crystal solubilization with Laemmli buffer, (2) size-based separation via SDS-PAGE, (3) electroelution of the target protein from the gel, and (4) dialysis to remove the elution buffer. From the *B. thuringiensis* AX isolate, four proteins ranging from ~20 to 60 kDa were recovered, but only AX-2 displayed cytotoxic activity toward MCF-7 breast cancer cells, while remaining non-hemolytic and non-toxic to normal cells (erythrocytes, PBMCs, and MRC-5 fibroblasts). Thus, AX-2 qualifies as a parasporin. AX-2 induces apoptosis in MCF-7 breast cancer cells without generating oxidative stress, and the observed cell death appears to initiate at the plasma membrane rather than through intracellular pathways.

## 1. Introduction

*Bacillus thuringiensis* (*B. thuringiensis*) is a Gram-positive, endospore-forming soil bacterium that produces crystalline δ-endotoxins known as Cry proteins [1]. These proteins are widely used as biocontrol agents against insects from several orders [1]. Interestingly, some Cry proteins that are non-toxic to insects exhibit marked cytotoxicity toward various mammalian cell lines, particularly human cancer cells [1]. These anticancer parasporal crystals have been classified as parasporins (PS) [2]. To standardize their nomenclature, the Parasporin Classification and Nomenclature Committee (http://parasporin.fitc.pref.fukuoka.jp/; accessed on 20 October 2025) established a taxonomically consistent quaternary ranking system based on amino-acid identity, analogous to the Cry toxin classification [3]. Six parasporin groups have been recognized to date, designated PS1 through PS6 [3,4].

At the end of the *B. thuringiensis* culture cycle, crystals (parasporal inclusions) coexist with spores, cellular debris, and extracellular medium. Consequently, purification requires sequential steps: separation of crystals by centrifugation, disruption of the crystal matrix through solubilization, and subsequent proteolytic activation of the released proteins [5,6,7,8]. During this process, solubilization liberates the protein components, and proteinase K digestion further fragments them into peptides of different sizes, which are typically tested as a mixture for cytotoxic activity against cancer cell lines.

A major challenge in cancer therapy is the limited selectivity of conventional drugs, which often damage healthy tissues and cause adverse effects that significantly impair patient quality of life [9]. This has driven intensive research toward alternative, naturally derived molecules capable of selectively targeting tumor cells. In this context, *B. thuringiensis*—widely distributed in nature and known for producing selective anticancer proteins—represents a promising source for the discovery of novel parasporins [2].

Although parasporins are formally defined as “*B. thuringiensis* and related parasporal proteins that are non-hemolytic yet preferentially cytotoxic to cancer cells” [10], many studies do not evaluate their effects on erythrocytes or non-cancerous cells. Furthermore, because parasporins are commonly obtained through protease digestion, identifying the specific peptide responsible for cytotoxicity is often impossible. To address these limitations, the present study employs a purification strategy that excludes protease treatment, enabling the recovery of intact proteins. In addition, the mode of cell death induced by the purified protein is examined while its effects on non-malignant cells are evaluated.

## 2. Results and Discussion

*B. thuringiensis* produces three types of parasporal crystals: Cry (insecticidal protein), Cyt (hemolytic protein), and PS (non-hemolytic but cytotoxic against cancer cells) [10,11]. Approximately 700 genes have been reported for Cry proteins (insecticidal) and 19 for different parasporins [3]. The crystal protein of each subspecies of *B. thuringiensis* showed significant differences in size and shape. Bipyramidal, spherical, cuboidal, rhomboidal, irregular crystals, and combinations of these structures have been observed [12]. These different morphologies may be due to genetic variations caused by differences in environmental conditions or habitat effects [13]. Therefore, it is important to perform a morphological, structural, and protein characterization of AX.

### 2.1. AX Parasporin Characterization

#### 2.1.1. PS-AX Morphological and Crystalline Analysis

**Figure 1:**
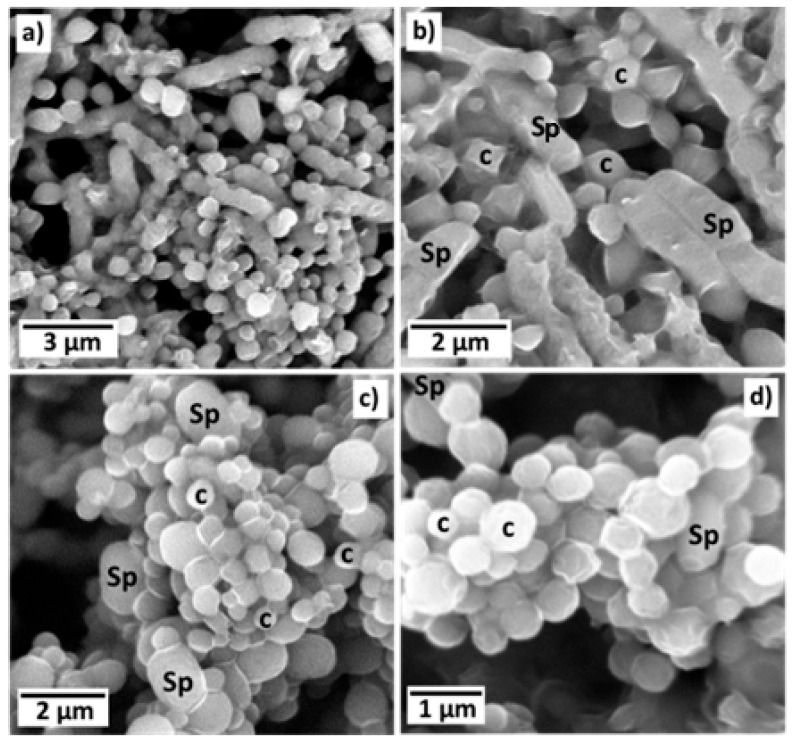
Scanning electron microscopy images of parasporal inclusions of AX-2 of *B. thuringiensis* AX strain from different regions and different magnifications (**a**–**d**). The images clearly show rod-shaped spores (Sp) and what could be identified as hemispherical and irregular crystals (c) both observed at (**b**–**d**).

**Figure 2:**
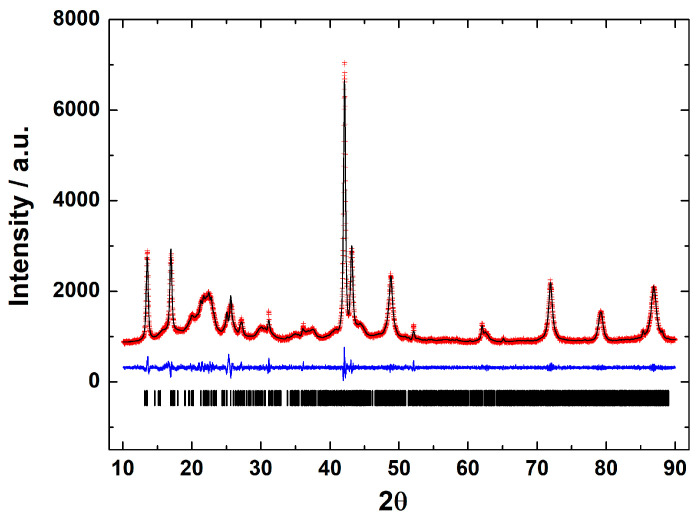
Le Bail fit of the X-ray powder diffraction pattern of the parasporal inclusions of *B. thuringiensis* AX strain. Black line shows the experimental pattern, red line is the theoretical pattern, and the blue line shows the difference (experimental—theoretical).

The morphology of parasporal inclusions of AX-2 of the *B. thuringiensis* AX strain were observed by SEM, as shown in Figure 1a–d. The images clearly show rod-shaped spores (Sp) and what could be identified as hemispherical and irregular crystals (c). The spores had an average size of approximately 2 × 1 μm, whereas the possible crystals were smaller than one μm. Identical or complex molecules are ordered in a regular tridimensional arrangement in crystals [14]. SEM results showed only the surface topography; therefore, to confirm that *B. thuringiensis* AX produces crystalline proteins, X-ray diffraction analysis must be conducted, as shown in Figure 2.

X-ray diffraction is a basic characterization technique that is normally used in material science to identify crystalline materials. For proteins, X-rays are used to solve their structures using a pure protein solution and recrystallizing it. Strangely, this technique is not used at all to detect the crystalline parasporal inclusion of *B. thuringiensis* from the culture media. A crystal is characterized by symmetry, with a limited number of unique molecular orientations imposed by the crystal lattice [14]. A typical powder diffraction pattern of the parasporal inclusions of *Bacillus thuringiensis* AX strain is shown in Figure 2. The diffraction pattern confirms that the objects observed in SEM are crystals. The crystals are monoclinic, space group P2_1_, with unit-cell parameters of a = 12.106(1) Å, b = 5.101(1) Å, c = 20.531(1) Å, β = 105.75°, and V = 1221.3(1) Å^3^. The lattice parameters obtained from the Le Bail fit of the protein from the *B. thuringiensis* AX isolate agreed with those reported for Cry1Ac (a bioinsecticide protein) from *B. thuringiensis* ssp. *kurstaki* HD-73 [15,16] confirming that the isolate produced crystalline bodies (Figure 2). Thus, crystalline parasporal inclusions of *B. thuringiensis* can be identified from culture media by X-ray diffraction using only crystallographic information from the literature (protein data banks), making it a rapid and powerful technique.

#### 2.1.2. PS-AX Protein Characterization

**Figure 3:**
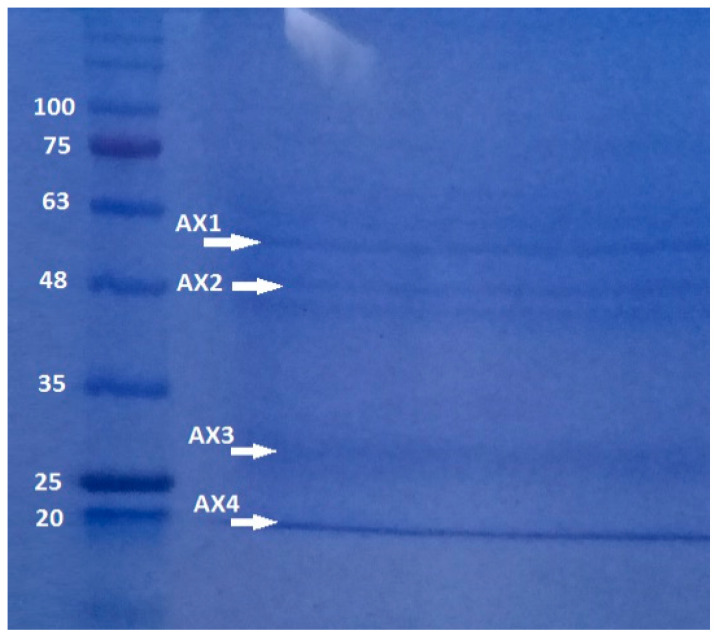
SDS-PAGE at 10% of proteins produced by *B. thuringiensis* AX strain isolated in the Papaloapan region in Oaxaca, México. Left Lane: molecular weight marker. Right Lane: parasporal inclusions, AX-1 ≈ 60 kDa, AX-2 ≈ 55 kDa, AX-3 ≈ 27 kDa, and AX-4 ≈ 20 kDa.

Polyacrylamide gel analysis shows that *B. thuringiensis* AX strain produces several parasporal proteins, although only four of them were selected according to their size and intensity: AX-1 (≈60 kDa), AX-2 (≈55 kDa) AX-3 (≈27 kDa), and AX-4 (≈20 kDa) (Figure 3). The protein concentrations after electroelution and dialysis were 0.17, 0.16, 0.34, and 0.6 mg/mL, respectively.

The quantity, concentration, and size of parasporal proteins in *B. thuringiensis* strains depend on many factors such as subspecies, culture media, operational conditions, etc. [17,18]. Similarly to the results of polyacrylamide gel analysis (Figure 3), other authors have reported the production of several insoluble proteins. Nair et al. [19] analyzed 18 isolated strains of *B. thuringiensis* cultivated on nutritive agar plates and incubated at 30 °C for 96 h. They observed that at least six parasporal proteins of different sizes and concentrations were produced by each isolate. Brasseur et al. [20] reported that *B. thuringiensis* 4R2, cultivated at 30 °C on nutrient agar at pH 7.1, produced five parasporal proteins, where the one at 37 kDa is identified as PS2Aa1.

**Figure 4:**
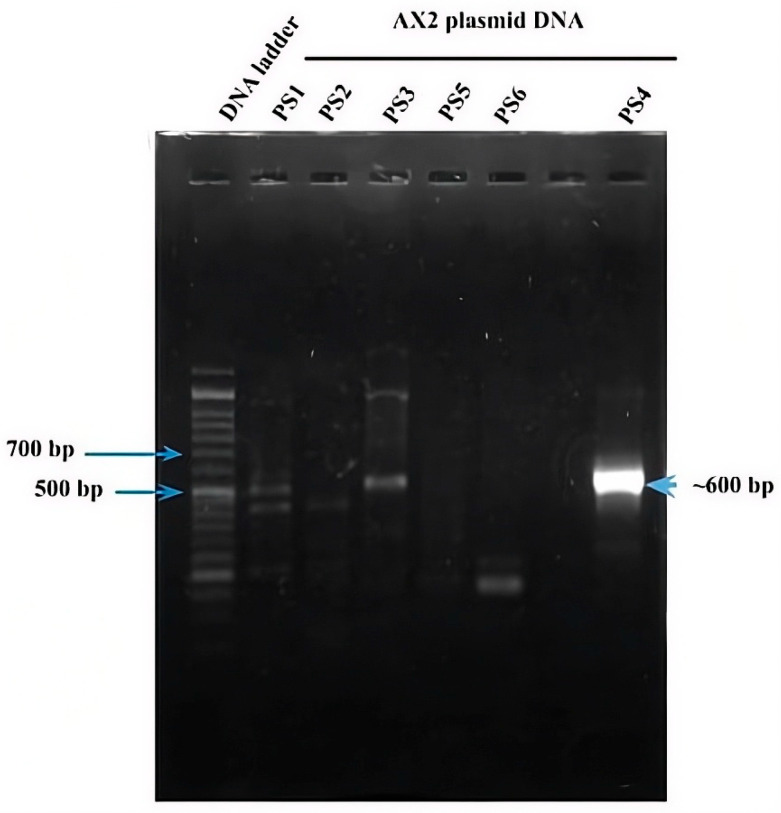
PCR amplification of the *ps1*–*ps6* genes from the AX strain. Line 1 DNA ladder. Lanes 2–6 and 8 correspond to the indicated PS family. Lane 7 has no sample.

**Figure 5:**
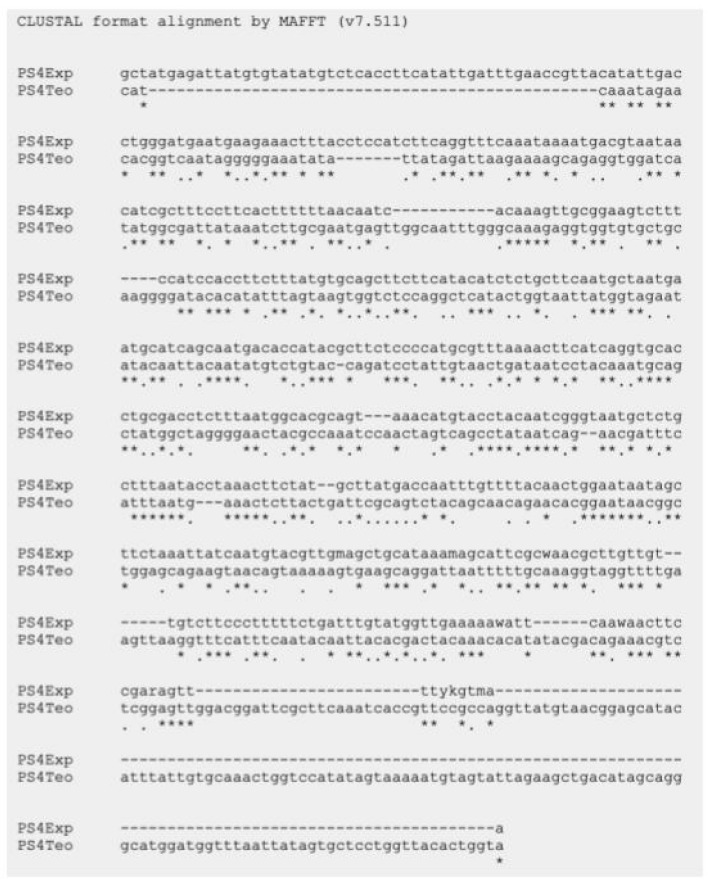
Sequencing analysis using MAFFT software of the ~600 bp nucleotide sequence of AX-2 purified protein obtained by PCR product and PS4Aa1. PS4Exp shows AX-2 amplicon sequence, PS4Teo shows reported PS4A1 sequence (NCBI accession number AB180980.2). * Indicate the homologous bases.

As mentioned above, six parasporin groups have been recognized to date, designated PS1 through PS6 [3,4]. Thus, to establish in which group AX-2 PCR belong, amplification of the PS1–PS6 genes from the AX strain was performed. PCR results for PS1 to PS6 showed nonspecific bands for each DNA amplicon, although PS4 showed a clear and specific amplification of ~600 bp, suggesting that AX-2 is a PS4 parasporin (Figure 4). Only two sequences of PS4 have been reported to this day: PS4Aa1 (NCBI accession number AB180980.2) and parasporin 4 (WP_029440439). The 600 bp amplification alignment using MAFFT software version 7 with reported PS4 sequences showed a low percentage of identity with PS4Aa1 (38%, Figure 5) and no significant similarity with parasporin 4 (Figure S1).

In *Bacillus thuringiensis*, genes encoding parasporins and Cry proteins are located in the DNA plasmid [21]. Limited information on the nucleotide sequence of parasporins generates uncertain identification results. According to Santos et al. [22] and the NCBI webpage, there are only eleven nucleotide sequences for PS1, three for PS2, two for PS3, two for PS4 (PS4Aa1 and parasporin 4), one for PS5, and one for PS6. Mizuki et al. [23] reported that their proteins have <25% homology to the existing Cry and Cyt proteins, assuming that is a new class of *B. thuringiensis* protein [23]. In other hand, Palma et al. [24], Crickmore et al. [25], and the Committee of Parasporin Classification and Nomenclature (http://parasporin.fitc.pref.fukuoka.jp/, accessed on 20 October 2025) reported a four-rank name depending on its degree of pairwise amino acid identity: first rank up to 45% similarity, second up to 78%, third up to 95%, and fourth higher than 95%, after the name of the protein (Cry, Cyt, and PS). Accordingly, PS1Aa1 and PS1Aa2 had 95% identity, and PS1Aa1 and PS1Ba1 had <45% identity [24,25]. Thus, although AX-2 showed a strong, clear, and specific amplification of PS4 (Figure 4), the identification with the two sequences reported was low (Figure 5), indicating that it could be a new class of parasporin or a new member of the PS4 parasporin family. However, further experiments are required to confirm this.

### 2.2. Cancer Cell Cytotoxicity of the Purified Parasporal Inclusions

**Figure 6:**
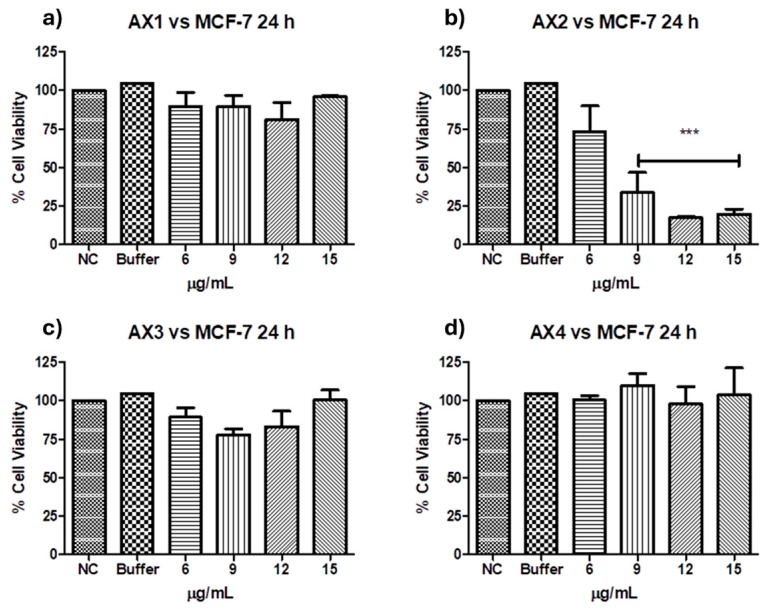
Cytotoxicity of *B. thuringiensis* AX strain parasporal inclusions against MCF-7 cells at 24 h of incubation (LC_50_ 7.5 µg/mL): (**a**) protein AX-1 ≈ 60 kDa, (**b**) AX-2 ≈ 55 kDa, (**c**) AX-3 ≈ 27 kDa, and (**d**) AX-4 ≈ 20 kDa. The vertical bars represent standard deviations. NC: control without treatment. Buffer: solution in which parasporal inclusions were dialyzed. Results are expressed as a percentage of the negative control (NC). Data are shown as mean ± standard deviation (SD). *** Represents *p* < 0.005 and it is considered statistically significant.

Antiproliferative activities against MCF-7 of the four parasporal inclusions produced by isolated *B. thuringiensis* AX were tested using the MTT technique, and the results are shown in Figure 6. No cytocidal activity against the MCF-7 cell line was observed for AX-1, AX-3, or AX-4 proteins (Figure 6a,c,d). However, AX-2 (≈55 kDa; Figure 6b) was highly cytotoxic to MCF-7 with a concentration between 12 and 15 µg/mL (17% of viability both), cytotoxic with 9 µg/mL (21% of viability), and moderately cytotoxic with 6 µg/mL (56% of viability). Brasseur et al. [20] reported ≈10% viability of MCF-7 cells treated with 5 µg/mL PS2Aa1 (37 kDa). The parasporal proteins produced by *B. thuringiensis* var. *kumamotoensis* J61 and *tohokuensis* J72 show 50% of the viability of MCF-7 treated with 1 and 2.79 µg/mL, respectively, even though the proteins purification process or the protein sizes are not clear [6]. Cry11a (66 kDa) of *B. thuringiensis* var. *israelensis* showed 90% viability of MCF-7 cells treated with 20 µg/mL [26].

At this point, only AX-2 showed cytotoxic activity against MCF-7 cells; thus, assays were performed using only AX-2 protein.

**Figure 7:**
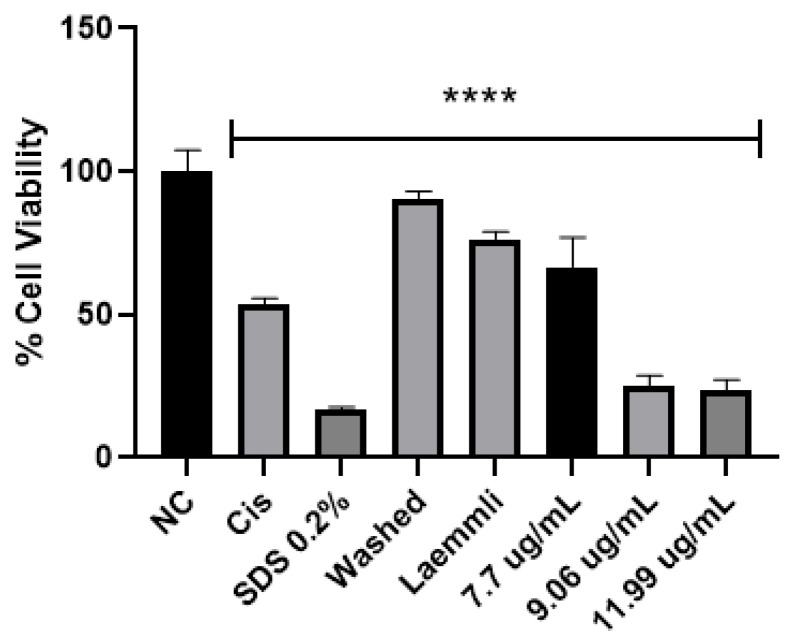
Cytotoxicity of AX crystal in MCF-7 cells before and after solubilization. The vertical bars represent standard deviations. NC: control without treatment. Cis: cisplatin and SDS 0.2% positive controls. Washed: sample before solubilization, ergo protein with a crystal structure. Laemmli: sample after solubilization, ergo protein without a crystal structure. 7.7, 9.06 and 11.9 µg/mL purified AX-2 protein. Results are expressed as a percentage of the negative control (NC). Data are shown as mean ± standard deviation (SD). **** Represents *p* < 0.0005 and it is considered statistically significant.

In the literature, parasporin crystals are separated from the culture media by centrifugation [5,6], broken by solubilization with Na_2_CO_3_-DTT-EDTA buffer [2,5,6,8,27], and finally activated by enzymatic treatment [2,5,6,7,8,27]. This process does not allow selectivity or control on enzyme digestion. Therefore, a four-step purification process was performed: (1) crystal solubilization with Laemmli buffer, (2) separation by molecular weight by SDS-PAGE, (3) electroelution to extract the selected protein from the polyacrylamide gel, and (4) dialysis to eliminate the elution buffer. The main advantages of this purification are that enzyme digestion does not break down the protein in the active or recognition site and toxic peptides can be identified. The viability test for parasporin with and without purification confirmed that purification was required to enhance the parasporin effect (Figure 7). In Figure 7, the washed sample is before and the Laemmli sample is after step 1, that is, the complete crystal and the free proteins. These results indicated that complete crystals and free proteins did not affect MCF-7 cell viability.

Two of the most common cancer drugs, doxorubicin (125 µg/mL) and cyclophosphamide (3125 µg/mL), reduce the viability of MCF-7 cells by approximately 10% and 35%, respectively [28]. Although cyclophosphamide markedly inhibits human lymphocyte (normal cell) proliferation, 10 and 40 µg/mL cyclophosphamide caused 35 and 55% cell death, respectively [29,30]. Moreover, it has been reported that MCF-7 cells become resistant to docetaxel and doxorubicin by increasing the concentration to 120 and 1000 nM, respectively [31]. Therefore, AX-2 cytotoxicity agrees with previous reports on parasporin, doxorubicin, and cyclophosphamide drugs.

### 2.3. Non-Cancer Cell-Toxicity of Purified Parasporal Inclusions

**Figure 8:**
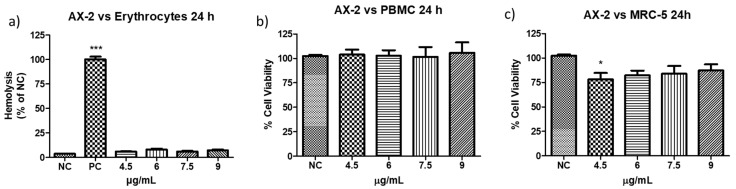
Toxicity of AX-2 against non-cancer cells: (**a**) erythrocytes, (**b**) peripheral blood mononuclear cells (PBMC), and (**c**) MRC-5 (fibroblasts). NC: control without treatment. Buffer: solution in which parasporal inclusions were dialyzed. Results are expressed as a percentage of the negative control (NC). Data are shown as mean ± standard deviation (SD). * Represents *p* < 0.05, *** *p* < 0.001 and were considered statistically significant.

The definition of parasporin is “*Bacillus thuringiensis* and related bacterial parasporal proteins that are non-hemolytic but capable of preferentially killing cancer cells” [10,18]. Therefore, to evaluate the toxicity of AX-2 against erythrocytes, peripheral blood mononuclear cells (PBMC), and MRC-5 (fibroblasts), further experiments were performed (Figure 8). The viability of erythrocytes and PBMC after treatment with AX-2 was not affected at any concentration (Figure 8a,b). For MRC-5 cells treated with AX-2, just 4.5 µg/mL is significantly different, affecting 20% of the cells (Figure 8c). These results indicated that AX-2 is non-hemolytic and preferentially kills cancer cells, ergo AX-2 is selective; therefore, AX-2 can be considered as parasporin.

Based on the definition of parasporin [10,18], several types of non-cancerous cells were treated with different *B. thuringiensis* parasporal crystal proteins. For example: normal uterus smooth muscle cells (UtSMC) with PS1Aa1 at a concentration from 0.78 to 25 µg/mL [2]; kidney epithelial cells extracted from an African green monkey (Vero) with BDzG protein at 15 to 50 µg/mL [32]; ovarian-surface epithelial cells (IOSE-144), human immortal endometrial stromal and epithelial cells (HIESC and HIEEC) and normal breast cells (MCF-10A) with PS2Aa1 at 0 to 20 µg/mL [20]; PBMC and embryonal kidney cells (HEK 293) with A13 protein at 0.02 to 12 µg/mL [18]; Vero cells with KAU protein at 0 to 100 µg/mL [33]; UtSMC, Vero, human chondrocytes (HC) with protein from A1547 isolate at 0.001 to 10 µg/mL [1]; normal T cells with 89-T-34-22 isolate proteins at concentrations up to 2 µg/mL [34]; MRC-5 and normal T cells with protein from 84-HS to 1-11 isolate at 0.01 to 100 µg/mL [23]; normal liver HC cells with CP78B protein at 0.1 to 10 µg/mL [4]; murine fibroblast cell line (NIH/3T3) HEK 293 and human corneal epithelial cell line (HCEC) with B.t.LDC 501 isolate proteins at 0.01 to 100 µg/mL [35]; keratinocyte cell line (HaCaT) with SLP isolate proteins at 0.25 to 1 µg/mL [36]; and human foreskin fibroblast cells (Hs27) with J61 and J72 isolate proteins at 0.1 to 20 µg/mL [6]. The protein concentration used in the treatment of non-cancerous cells was related to the concentration used to evaluate the viability of cancer cells. Moreover, each parasporal crystal protein showed toxicity against several cancer cell lines at different concentrations. Therefore, the protein concentrations used are diverse, and it is pointless to perform a comparative analysis. However, all those authors reported non- or minimal toxicity (up to 80% viability) for proteins against normal cells. Our results showed that PBMC and MRC-5 treated with AX-2 (4.5 to 9 µg/mL) had minimal toxicity at one concentration (4.5 µg/mL in MRC-5) and non-toxicity at the other concentrations (Figure 8).

Parasporins must be non-hemolytic but cytotoxic against cancer cells [10,37]. Accordingly, it has been reported that from 1744 *B. thuringiensis* strains evaluated, 3.4% of them produced a parasporal protein that induced a strong hemolysis, and 2.4% produced a protein with non-hemolytic but cytotoxic properties against cancer cells [38]. In another study, 0.32% of 1837 strains formed a hemolytic protein, 4.4% a non-hemolytic protein, and 29.6% parasporins. Moazamian et al. [5] tested 88 *B. thuringiensis* strains, and 85% were non-hemolytic and 4.5% produced parasporins. Therefore, it is important to perform both hemolytic and cytotoxicity analyses to identify parasporins.

There is no consensus in the parasporin literature regarding the acceptable hemolysis level. Ishii and Ohba [39] defined one unit of hemolytic activity as the activity required to increase the absorbance at 540 nm by 0.1 after 18 h incubation at 27 °C. Their proteins showed from 0 to 160 hemolytic units. Mizuki et al. [23] defined high hemolytic activity at >1.00 of absorbance, moderate from 1 to >0.5, low from 0.5 to >0.2, and non-hemolytic at ≥0.2. Moazamian et al. [5] defined an absorbance > 0.20 as non-hemolytic activity. Aberkane et al. [32,40] defined as no hemolytic activity a 0.2 absorbance, slightly significant hemolysis between 0.2 and 1 of absorbance, and from ten proteins tested just two showed >0.2 absorbance. Aljeldah et al. [41] used API biochemical tests to identify non-hemolytic strains and reported only one non-hemolytic strain. Other authors reported a percentage of hemolysis of 9.2 to 23% [38] and from 4.6 to 32.4% [42], or only reported as non-hemolytic or strong hemolytic activity [18,43].

Hemolysis is the most used method to evaluate the toxicity of intravenously administered therapies [44]. Moreover, to characterize novel compounds intended for interactions within human biological systems in vivo, the initial step is the hemolysis assay [45]. Hemolysis is the rupture of red blood cells, followed by the release of intracellular material into the bloodstream, and its consequences include anemia, icterus, and hemoglobinuria [46]. According to Figure 8a, AX-2 at the tested concentration is non-hemolytic, as it is shown from the low hemolysis percentage 5 to 8% (0.11 to 0.16 of absorbance). Therefore, parasporal protein AX-2 is non-hemolytic and a potential molecule for intravenous administration.

### 2.4. Morphological Changes and Cell-Death Mechanism Induced by Parasporal Protein AX-2

**Figure 9:**
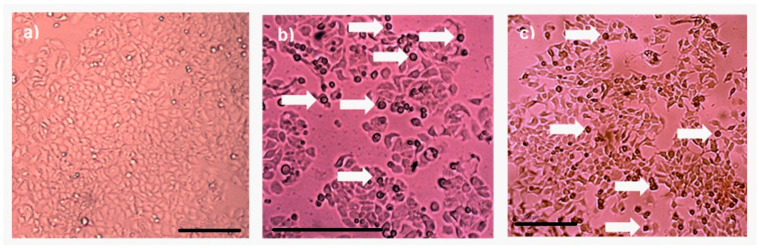
Cytopathic effect of parasporal inclusion AX-2 of *B. thuringiensis* AX strains against MCF-7 cells. Inverted microscopic observations were performed 24 h post-inoculation: (**a**) negative control, (**b**) positive control (doxorubicin 8 µM), and (**c**) AX-2 parasporal inclusion treatment. The arrows indicate apoptotic bodies, and the scale bar is 100 μm.

Protein AX-2 complies with the parasporin definition because it is non-hemolytic and cytotoxic against cancer cells but non-cytotoxic against normal cells (Figure 6 and Figure 8). The next step is to study the type of death it causes. Morphological changes are associated with damage in the cells; thus, a microscopic analysis of MCF-7 cells with parasporal protein AX-2 treatment was performed with 9 µg/mL at 24 h. The cytopathic effects are shown in Figure 9.

No detectable cytopathy was observed in the negative control (Figure 9a) cells, which showed a normal angular or polygonal shape. As expected, doxorubicin samples showed signs of apoptosis, such as reduced cytoplasm, chromatin condensation, and the formation of apoptotic bodies (Figure 9b). The parasporal protein AX-2 on MCF-7 cells after 24 h of incubation exhibited the typical irregular morphology of cells in apoptotic processes (Figure 9c). The cells showed round or oval dark masses associated with nuclear chromatin fragments. The morphology had an almost normal polygonal shape with marked lesions that appeared on the surface of the plasma membrane, indicating apoptotic cell death (Figure 9b,c). Thus, morphological changes induced by AX-2 indicated apoptotic cell death (Figure 9). Similar morphologies have been observed in cancer cells treated with parasporins in MCF-7 cells [18,20], PC-3 and HepG2 [20], Hep2 and A549 [32], and HeLa [33].

### 2.5. Flow Cytometric Analysis by Annexin V/PI Assay and Trypan Blue Staining

**Figure 10:**
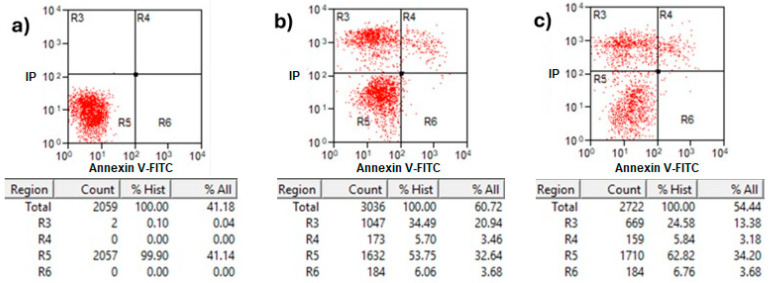
Apoptosis and necrosis determination of treatment with AX-2 protein for 24 h of incubation on MCF-7 cells: (**a**) negative control cells, (**b**) positive control, and (**c**) AX-2. Annexin V/PI flow cytometry where R3 expresses necrotic cells, R4 late apoptotic cells, R5 viable cells, and R6 early apoptotic cells after AX-2 protein treatment at 9 µg/mL.

Annexin V/propidium iodide (PI) staining analysis of treated MCF-7 cells with protein AX-2 is shown in Figure 10. Healthy cells in the R5 quadrant with intact membranes remain Annexin V-negative and PI-negative (Annexin V^−^/PI^−^). Early apoptotic cells in the R6 quadrant expose phosphatidylserine (PS) on their outer membrane, which is bound by Annexin V, resulting in Annexin V-positive/PI-negative cells. Late apoptotic in quadrant R4 and necrotic cells in R3 have damaged membranes, allowing both Annexin V to bind the exposed PS and PI to enter and bind DNA, creating Annexin V-positive/PI-positive cells [47]. The results showed that 24 h after treatment, the parasporal inclusions AX-2 of the *B. thuringiensis* AX strain induced elevated levels of necrosis in MCF-7 cancer cell lines (Figure 10). These results are like those obtained from the MTT assay (Figure 6). Approximately 72% of the dead cells showed an elevated level of necrosis after 24 h, and, in 18% of the deaths of the cells, AX-2 induced apoptosis. The results from flow cytometric analysis (Figure 10) indicate major events in the necrosis quadrant (R3) and minor events in late apoptosis (R4).

**Figure 11:**
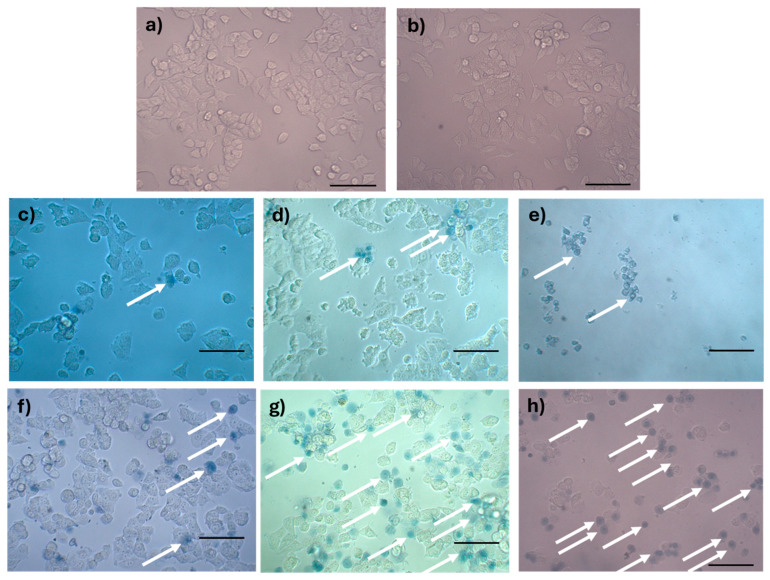
Analysis of rupture of the plasma membrane by trypan blue staining of MCF-7 cells treated with AX-2: (**a**) negative control, (**b**) tris-glycine buffer, (**c**) doxorubicin (positive control for apoptosis), (**d**) cisplatin (positive control for apoptosis), (**e**) sodium dodecyl sulfate (positive control for sudden death), (**f**) 1 h of AX-2 treatment, (**g**) 3 h of treatment, and (**h**) 5 h of treatment. The arrows indicate the cells with damaged membranes. The scale bar is 100 μm.

Parasporins trigger apoptosis in several cancer cell lines, including MDA-MB-231 [41], Jurkat [48], MCF-7 [23,40], HepG2, PC-3 [40], SW480, and CaCo-2 [40]. Some of these references show results in Annexin V–PI flow cytometry with counts in the R3 quadrant (Annexin V^−^/PI^+^ for necrosis). The Annexin V/PI assay results indicated necrosis (Figure 10); however, morphological analysis indicated apoptosis based on reduced cytoplasm, apoptotic bodies formation, and chromatin condensation (Figure 9). Comparable results were obtained by Brasseur et al. [20] for PC-3, MCF-7, and HEPG2 cells treated with PS2Aa1 parasporin. Because many cells are PI-positive in Annexin V–PI flow cytometry, the authors decided to perform a caspase-3/7 assay to check if PS2Aa1 activates it and induces apoptosis. Suarez-Barrera et al. [40], Amano et al. [48] and Aljeldah et al. [41] also confirmed this by measuring caspase activity. In contrast, Costigan et al. [49] reported that Annexin V staining does not discriminate between apoptotic and necrotic cells when plasma membrane permeabilization occurs. Therefore, to investigate further the cell-death mechanism, trypan blue staining was performed to evaluate membrane damage (Figure 11).

Trypan blue staining results are shown in Figure 11. The negative control without AX-2 treatment showed cells with a normal angular or polygonal shape (Figure 11a). The tris-glycine (TG) buffer is the protein vehicle, and, ergo, is where AX-2 is dissolved. MCF-7 cells treated with TG showed a normal angular or polygonal shape (Figure 11b), indicating that TG did not induce cytotoxicity in the cells. Doxorubicin and cisplatin treatment showed signs of apoptosis, such as cytoplasm and chromatin condensation and the formation of apoptotic bodies, as expected (Figure 11c,d). The effect of sodium dodecyl sulfate (SDS) was sudden, breaking the cell membranes. SDS is normally used to lyse MCF-7 cells [50]. The lipid bilayer of MCF-7 cells is damaged, and the internal components are affected by SDS, which prevents staining with trypan blue, as this results in a sudden death. The morphological changes caused by AX-2 in MCF-7 cells were typical of apoptosis, that is, cell shrinkage, compaction of internal structures, and the formation of round bodies (Figure 11f–h). These results indicate that AX-2 increases membrane permeability, which translates to an apoptosis death. Moreover, membrane damage was observed after the first hour of AX-2 treatment and increased with time. After 5 h of treatment, the typical MCF-7 cell morphology was completely lost, indicating that the AX-2 mode of action is directly associated with an interaction with the cell membrane.

**Figure 12:**
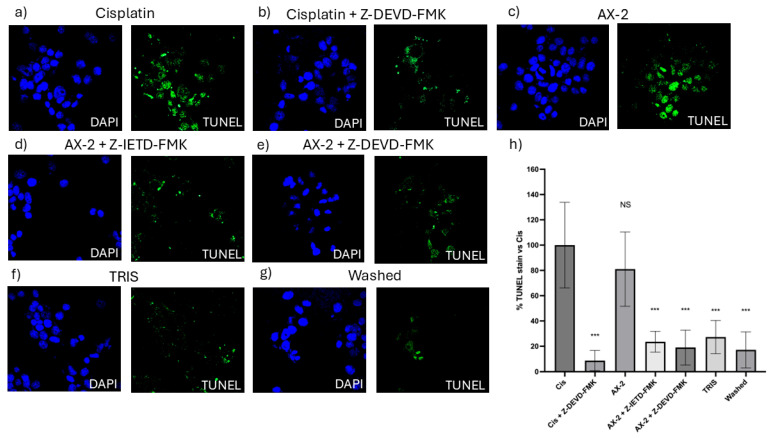
TUNEL micrographs (**a**) positive control (cis; cisplatin); (**b**) cisplatin with caspase 9 inhibitor (Cis + Z-DEVD-FMK); (**c**) AX-2 protein purified; (**d**) AX-2 with caspase 8 inhibitor (AX-2 + Z-IETD-FMK); (**e**) AX-2 with caspase 9 inhibitor (AX-2 + Z-DEV-FMK); (**f**) negative control (TRIS); (**g**) protein before crystal solubilization (washed); and (**h**) cell labeling expressed as a percentage of the negative control. Data are shown as mean ± standard deviation (SD). *** Represents *p* < 0.001 and it is considered statistically significant. NS is not statistically significant.

To confirm cellular death induced by AX-2, a TUNEL assay was performed (Figure 12). The terminal deoxynucleotidyl transferase dUTP nick-end labeling (TUNEL) assay identifies nicks or breaks in DNA by labeling the exposed 3′-OH ends of fragmented DNA strands [51]. Positive TUNEL staining indicates that the cell nucleus is undergoing degeneration and is likely to undergo apoptosis owing to nuclear DNA fragmentation [52]. To validate the TUNEL technique, caspase 8 and 9 inhibitors were used to turn off the apoptosis process. AX-2 treatment was not significantly different from the positive control; AX-2 with caspase 8 and 9 inhibitors, the sample before crystal solubilization, and cisplatin with a caspase 9 inhibitor showed no significant difference from the negative control (Figure 12). These results corroborate that AX-2 induces apoptosis and that the protein before crystal solubilization (washed) does not induce apoptosis in MCF-7 cells.

The results so far show that AX-2 changed MCF-7 cell morphology to a round or oval dark mass, associated with nuclear chromatin fragments, with lesions on the surface of the plasma membrane (Figure 9), induced cell shrinkage and internal structure compaction (Figure 11), and DNA fragmentation (Figure 12). These results indicate that AX-2 induced apoptosis in MCF-7 cells. In addition, trypan blue staining results suggest that purified AX-2 mode of action might be directly associated with cell-membrane interactions. This is supported by the lack of a complete crystal effect on MCF-7 cells (Figure 7 and Figure 12). Rezaei et al. [53] identify the parasporin produced by *B. thuringiensis* E8 as a PS4Aa1 cytotoxic for MCF-7 cells. Their proteins increase plasma-membrane permeability and damage the cell membrane, leading to apoptotic mechanisms. The authors proposed that PS4Aa1 exerts its effects through a specific receptor on the cell membrane of cancer cells. Although our results coincide with those of Rezaei et al. [53], during the AX-2 purification process, Laemmli buffer is used to denature the protein.

Refolding proteins is a common laboratory and industrial process, especially in the production of recombinant proteins [54]. In general, recombinant protein recovery begins with isolation, followed by solubilization, refolding, and purification. Solubilization is conducted with strong anionic detergents such as sodium dodecyl sulfate (SDS), which denatures the protein [54]. Detergents attached to the protein favored and stabilized its denatured form, because it reduced the free energy for denaturation and increased the free energy for protein aggregation; therefore, it needs to be removed or diluted. Conversely, to refold the protein, the free energy of denaturation must be increased and the free energy of association must be decreased [54]. This is achieved using osmolytes as stabilizers, which include sugars and polyhydric alcohols, such as glycerol. Other reagents used to refold proteins are protein aggregation inhibitors, such as Tris, PEG, CHAPS, DMSO, and MES [54]. During AX-2 solubilization, separation by molecular weight, and electroelution, the sample contained SDS that was removed during dialysis, and the samples were stored in Tris-glycine buffer at 5 °C. During dialysis, SDS is removed so the free energy for protein aggregation is increased. Then, during storage, the protein is diluted in Tris-glycine buffer, and this could stimulate the protein to be refolded. Although, future experiments must be performed to unravel the AX-2 possible refolding mechanism. Even the folding state of the protein remains uncertain, and the data presented in Figure 6, Figure 7, Figure 11 and Figure 12 clearly indicate that the purified protein displays cytotoxic activity leading to apoptosis.

To determine whether AX-2 treatment generated oxidative stress in normal cells (PBCM and MRC-5) and MCF-7 cells, reactive oxygen (ROS) and nitrogen species (NO) levels were measured.

### 2.6. Oxidative Stress

**Figure 13:**
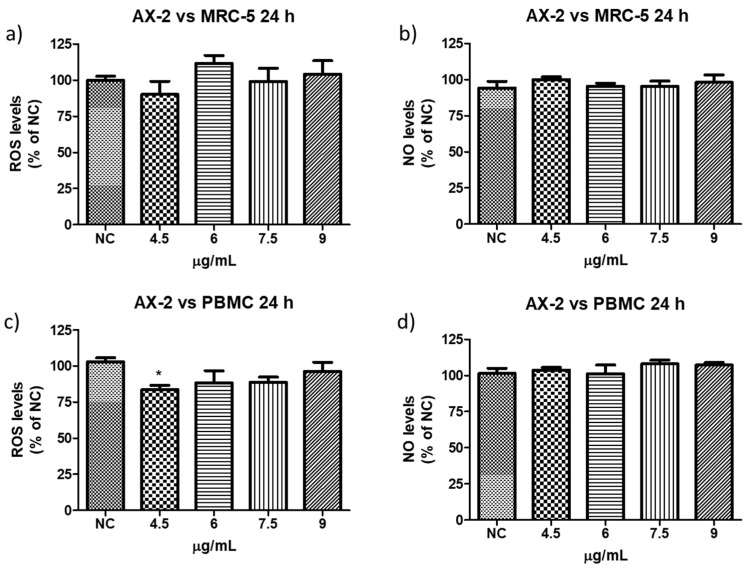
Reactive oxygen and nitrogen species production following AX-2 treatment in normal cells: (**a**) reactive oxygen species in MCR-5 cells, (**b**) reactive nitrogen species in MRC-5 cells, (**c**) reactive oxygen species in PBCM cells, and (**d**) reactive nitrogen species in PBCM cells. NC: control without treatment. Results are expressed as a percentage of the negative control (NC). Data are shown as mean ± standard deviation (SD). * represent *p* < 0.05 and was considered statistically significant.

Reactive oxygen species (ROS) are a group of highly reactive molecules such as hydroxyl, superoxide free radicals, and hydrogen peroxide [50]. Moderate ROS levels are important for the regulation of cell proliferation and differentiation; however, excessive ROS production can promote cell death [50]. Therefore, reactive oxygen and nitrogen species (NO) levels were measured to determine whether AX-2 treatment generated oxidative stress in normal cells (PBCM and MRC-5) (Figure 13). AX-2 generated a slight change in ROS and NO levels in MRC-5 cells and PBMC, although no statistical significance was obtained (Figure 13a,b,d). Except for 4.5 µg/mL of AX-2 against PBMC, the ROS level decreased by 15% (Figure 13c).

**Figure 14:**
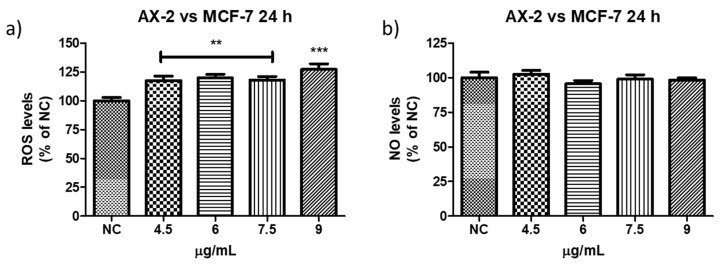
Reactive oxygen and nitrogen species production after AX-2 treatment in MCF-7 cells: (**a**) reactive oxygen species and (**b**) reactive nitrogen species. NC: control without treatment. Results are expressed as a percentage of the negative control (NC). Data are shown as mean ± standard deviation (SD). ** Represents *p* < 0.005, *** *p* < 0.0005 and were considered statistically significant.

ROS have been reported to induce various biological processes including apoptosis [55]. Moreover, various anticancer agents induce cytotoxicity in cancer cells by increasing ROS production [50]. Reactive oxygen and nitrogen species were measured to establish whether AX-2 treatment generated oxidative stress in MCF-7 cells and triggered apoptosis (Figure 14). AX-2 treatment did not affect NO levels in MCF-7 cells (Figure 14a). However, AX-2 treatment slightly increased ROS levels to 20% (Figure 14b).

There are few contradictory studies on ROS and NO levels in parasporins. Borin et al. [18] treated MCF-7 with A13-2 (4–12 µg/mL) and observed an increment of 24% in NO levels but no changes in ROS levels. The authors indicated that the increase in NO was not sufficient to trigger oxidative stress. Aljeldah et al. [41] reported that parasporin from HAU-145 isolate elicited greater levels of ROS (29.4%) in MDA-MB-231 cells. The authors concluded that the cytotoxic effect of parasporin is associated with the induction of apoptosis through ROS. Krishnan et al. [56], for HepG2 treated with Cry1Aa (10 µg/mL) and Cry1Ca (10 µg/mL), reported no significant increment in ROS levels from ≈1 × 10^5^ to ≈2 × 10^5^ relative fluorescence units (RFU), when compared with the positive control. Accordingly, the RFU was duplicated and was considered to have no significant increment in ROS levels. In contrast, the use of other molecules against MCF-7 cells provides more cohesive information. Huynh and Heo [50] treated MCF-7 cells with phorbol myristate acetate (PMA, 50 nM) and observed an increase of approximately 200% in ROS production. When MCF-7 cells were treated with ginsenoside Rh1 (5–50 µM) and PMA, the ROS levels increased to approximately 300%. The authors indicated that treatment with PMA slightly increased ROS levels in MCF-7 cells, whereas treatment with Rh1 and PMA significantly enhanced ROS production. Cao et al. [55] treated MCF-7 cells with surfactin (30 µM) during 1–9 h and observed that ROS levels were increased from ≈150 to 530%. The authors assumed that the increase in ROS production due to surfactin treatment was related to surfactin-induced apoptosis in MCF-7 cells. Mahalingaiah et al. [57] used a source of ROS at doses of 25 µM and 250 µM to study the oxidative stress effects on the growth, survival, and tumorigenic potential of MCF-7. The lower dose generated ≈ 150% of ROS and the higher dose ≈ 275% of ROS. The lower dose resulted in a statistically insignificant decrease in MCF-7 cell growth. According to these studies, the minimum ROS level required to generate oxidative stress is 150%. Therefore, as shown in Figure 13 and Figure 14, ROS levels were not sufficient to generate oxidative stress in PBMC and in MCF-7 cells, respectively, with AX-2 treatment.

Six parasporin groups have been recognized to date, designated PS1 through PS6 [3,4]. PS1 proteins are three-domain toxins, similar to Cry proteins. Evidence suggests that PS1 interacts with the Beclin-1 receptor, thereby triggering apoptotic cell death [22]. Members of the PS2 family are pore-forming toxins classified within the β-PFT subgroup. Previous studies indicate that PS2 proteins are likely to induce apoptosis, which is associated with upregulation of the tumor-suppressor gene PAR-4 and inhibition of the PI3K/AKT signaling pathway [22]. PS3 proteins also belong to the three-domain toxin group; however, their mechanism of action differs, as they induce cell death primarily through necrosis by forming pores in the plasma membrane and increasing membrane permeability [22]. PS4 proteins share structural similarities with PS2 and are likewise classified as β-PFTs, but their cytotoxic mechanism resembles that of PS3, leading to necrotic cell death. These proteins bind nonspecifically to the target cell membrane and assemble into oligomeric pore complexes that disrupt membrane integrity [22]. PS5 is also a member of the β-PFT subgroup, although its mechanism of action has not yet been elucidated. PS6 proteins are structurally related to PS1 (three-domain type), but their mode of action remains unknown [22].

Cytometry (Figure 10) supports PCR amplification (Figure 4) in terms of the fact that the AX-2 protein is a member of the PS4 family. On the other hand, cytopathic effect (Figure 9), trypan blue staining (Figure 11), and TUNEL (Figure 12) results indicated that the AX-2 mode of death is apoptosis, like PS1 or PS2. Nevertheless, the identity of AX-2 with reported PS4Aa1 is only 38% (Figure 5), while no significant similarity was found with reported parasporin 4 (Figure 1a). These results support the hypothesis that AX-2 could be a new class of parasporin. However, further experiments are needed to corroborate this hypothesis.

Every year, the cancer incidence increases dramatically, and its detection at advanced stages makes effective treatment difficult. In this sense, the small protein AX-2 (≈55 kDa), with high specificity and effectiveness in killing cancer cells, is a strong candidate for breast cancer treatment. The electrostatic characteristics of small proteins are important for their interactions with cancer cell membranes, which usually lead to irreversible damage [58]. The plasma-membrane composition of cancer cells makes them more susceptible to small proteins, such as AX-2, allowing for their specific recognition [59,60]. Likewise, structural changes in the cancer cell membrane increase the contact area, which enhances their interaction with small proteins [61]. Moreover, some membrane-receptors are overexpressed in cancer cells, which enhances protein and membrane interactions [42]. Annexin/IP (Figure 10) and trypan blue staining (Figure 11) results clearly demonstrate early membrane damage due to the rapid and efficient interaction of AX-2 with MCF-7 cells, unlike the results for non-cancerous cells, where no damage was observed. This membrane-level damage can trigger molecular events that lead to the death of neoplastic cells through apoptosis or necrosis. One of the most important characteristics of apoptosis is the controlled morphological changes in cell structure. Unlike apoptosis, necrosis cell death is rapid and uncontrolled, leading to spillage of intracellular contents [62]. Optical microscopy analysis (Figure 9) indicated typical morphological changes in death by apoptosis in the first hour of treatment with AX-2, i.e., apoptotic bodies were detected. Reactive oxygen and nitrogen species can induce apoptosis in cancer cells [63,64,65]. The results showed that the plasma-membrane disruption generated by AX-2 treatment led to apoptosis in MCF-7 cells, independent of ROS and NO production (Figure 13 and Figure 14). In summary, the cytotoxic effect of AX-2 results from membrane damage that causes early apoptosis, which leads to cell death in the MCF-7 cell line.

## 3. Materials and Methods

### 3.1. Bacterial Strains and Culture Conditions

The *Bacillus thuringiensis* strain AX used in this study was isolated from the Papaloapan basin region [66]. Bacterial cells were grown at 30 °C in nutritive broth at 180 rpm for seven days. The end-of-culture criterion was set to 85–90% of the released endospores determined microscopically [17]. The culture medium was centrifuged at 5500 rpm for 20 min at 4 °C and washed with distilled water (once), 0.85% *w*/*v* NaCl (three times), distilled water (once), acidified water (pH 2.5; three times), and distilled water (once). For every wash, the pellet was resuspended in 5 mL of the solution and centrifuged at 5500 rpm for 20 min at 4 °C. After washing, the pellet was resuspended in 5 mL distilled water [17].

### 3.2. Parasporal Protein Purification

The protein purification process was performed in four stages: (1) crystal solubilization by adding Laemmli buffer [67] (5 mL Tris pH 6.8 buffer, 2 mL SDS 10% *w*/*v*, 2 mL glycerol, 1 mL 2-mercaptoethanol and 1 mL bromophenol blue 1% *w*/*v*) at a 1:2 sample/buffer ratio and heating in boiling water for 5 min, (2) separation by molecular weight and selection of the band, (3) extraction of the selected protein from the sodium dodecyl sulfate polyacrylamide gel electrophoresis (SDS-PAGE) gel and elution of the selected protein from the gel slice, and (4) separation of the protein from the elution buffer. Separation by molecular weight was performed by SDS-PAGE at 10%, with molecular weight markers (11 to 96 kDa, NZYTech LMW Protein Marker II; NZYTech, Lisbon, Portugal) and subjected to a constant voltage of 100 V. Proteins were selected according to their molecular weights (≈20, 27, 55, and 60 kDa). The bands with the selected proteins were cut from SDS-PAGE and placed in a 1 mL microtube with tris-base + glycine buffer (TB + G; 3 g + 15 g, respectively, +1 L of distilled water). These samples were eluted at 10 mA for 200 min in an Electro-Eluter (Model 422, Bio-Rad, Hercules, CA, USA). The elution buffer (TB + G) and SDS from the electrophoresis gel are toxic to cancer cells. Hence, dialysis was performed with D6191 Dialysis sack MWCO: 12,000 Da (Sigma-Aldrich, St. Louis, MO, USA) at 4 °C for 24 h using Tris base-Glycine Buffer (Tris base 3 g/L; glycine 14.4 g/L in deionized water) and changed every two hours. Protein concentration was estimated using the Bradford method [68] at 595 nm, with bovine serum albumin (BSA) as the standard. As a control for the purification process, the cytotoxicity of AX with crystal structure (before crystal solubilization; washed) and without crystal structure (after crystal solubilization; Laemmli) was tested against MCF-7 cells.

### 3.3. Cells and Culture Conditions

MCF-7 (human breast cancer cells) and MRC-5 cells (human fetal lung fibroblast cells) were acquired from American Type Culture Collection ATCC, Manassas, VA, USA (ATCC^®^ HTB-132™; and ATCC^®^ CCL-171™, respectively).

The human breast cancer cell line MCF-7 was maintained in Dulbecco’s modified Eagle’s high glucose medium (DMEM, Biowest, Nuaillé, France). Non-cancerous cells (erythrocytes, peripheral blood mononuclear cells, and fibroblasts) were maintained in Roswell Park Memorial Institute Medium (RPMI 1640, Biowest, Nuaillé, France) at a pH of 7.4. Both media were supplemented with 10% fetal bovine serum (FBS; Biowest, Nuaillé, France), 100 U/mL penicillin, 100 μg/mL streptomycin, and 2 mM L-glutamine (Biowest, Nuaillé, France). All the cells were incubated at 37 °C in a humidified incubator with 5% CO_2_. To harvest adherent cells, the growth medium was removed, and the cells were washed with phosphate-buffered saline (DPBS, Biowest, Nuaillé, France). To produce a cellular suspension, a cell-dissociation solution composed of trypsin 0.25% and EDTA was added and incubated at 37 °C for 3 min in a humidified 5% CO_2_ incubator. Trypsinized cells were re-seeded in fresh medium at 10^5^ cells/mL and incubated at 37 °C in a humidified 5% CO_2_ incubator.

### 3.4. Cytotoxicity Assay by MTT

The cytotoxicity of parasporal inclusions was evaluated using the 3-(4,5-Dimethylthiazol-2-yl)-2,5 diphenyltetrazolium bromide (MTT) assay [69]. MCF-7 cells (2 × 10^4^ cell/100 μL) were cultured in 96-well microplates (TPP) with DMEM high glucose and RPMI 1640 and incubated at 5% CO_2_ for 24 h with different concentrations of protein. After incubation, 10 μL MTT (5 mg/mL, Sigma-Aldrich, St. Louis, MO, USA) dissolved in PBS was added and incubated for 4 h. At the end of this period, the medium was removed, and insoluble formazan crystals were dissolved in 100 μL of dimethyl sulfoxide (DMSO). The optical density produced by this product was measured at a wavelength of 595 nm using a microplate reader (Bio-Rad iMark, Hercules, CA, USA). Each treatment was performed in triplicate and repeated three times. The negative controls used were no treatment, the buffer where protein was dissolved (PBS), and the positive controls were cisplatin and SDS 0.2%.

The cytotoxicity of the parasporal inclusions against non-cancerous cells was evaluated using the same conditions as the MTT assay for cancer cells. Non-cancerous cells include erythrocytes, peripheral blood mononuclear cells (PBMC), and fibroblasts (MRC-5). Erythrocytes and PBMCs were donated by the Clinical Analysis Laboratory of Franciscan University (LEAC-UFN). The LEACUFN laboratory obtained the cells from blood samples discarded from healthy adults (experimental protocols used were approved by the UFN Ethics Committee on Human Beings; CAAE number: 31211214.4.0000.5306) in the absence of identification data. All procedures followed the ethical standards of the institutional and/or national research committee and the 1964 Declaration of Helsinki. According to the UFN Ethics Committee on Human Beings, signed informed consent was not required. The samples were obtained by venipuncture using Vacutainer^®^ with heparinized tubes and were separated using a concentration gradient with Histpoaque^®^-1077 (Sigma-Aldrich, St. Louis, MO, USA) by centrifugation, following the method described by Sagrillo et al. [70]. The cells were counted using a Neubauer hemocytometer.

### 3.5. Light Microscopic Analysis

MCF-7 cell morphological changes induced by parasporal proteins incubated for 24 h were analyzed by light microscopy. The cytopathic effect was monitored using an inverted microscope (Motic AE31E, Moticam 5 Plus., Richmond, BC, Canada). The images were captured and analyzed for cell morphology using Motic images plus 3.0 software. Doxorubicin (8 µM) and no treatment were used as the positive and negative controls, respectively.

### 3.6. Flow Cytometric Analysis by Annexin V/PI Assay

The FITC Annexin V/Dead Cell Apoptosis Kit (Molecular Probes Inc., Eugene, OR, USA) was used according to the manufacturer’s instructions. Annexin V binds to phosphatidylserine on the outer leaflet of the plasma membrane, and its presence on the outer leaflet is a unique feature of early apoptosis. Propidium iodide (PI) binds to DNA from cells with disrupted cell membranes in late apoptosis and necrosis and is excluded from cells with intact membranes [71]. Cells incubated for 24 h with parasporal protein were collected, washed with PBS, and diluted in 1X annexin binding buffer (100 μL). For each sample, 5 μL of Annexin V and 2 μL of PI were added to the cell suspension and incubated for 15 min at room temperature. An additional 100 μL of annexin binding buffer was added to each sample for a total of 200 μL. Samples were analyzed (15,000 events) using a BD FACSAria flow cytometer, and the analysis was performed using BD FACSDiva v8.0 software (Becton, Dickinson and Company, Franklin Lakes, NJ, USA). The negative control used was no treatment, and the positive control was doxorubicin (1 µM).

### 3.7. Trypan Blue Staining

Some promising anticancer molecules alter the membrane permeability and lipid composition [72,73]. Trypan blue labeling is restricted to dead cells due to plasma membrane rupture, which is correlated with trypan blue staining intensity [74]. Dead cells absorb trypan blue, and the cytoplasm is stained owing to the loss of membrane integrity, while viable cells remain unstained.

For trypan blue staining, MCF-7 cells (2 × 10^4^ cell/100 μL) were cultured in 96-well microplates with DMEM high glucose and RPMI 1640 and incubated at 5% CO_2_ for 24 h with different concentrations of protein. After that, AX-2 (80 µg/mL), tris-glycine buffer (25 mM tris base and 192 mM glycine), sodium dodecyl sulfate (SDS, 1%), doxorubicin (6 µg/mL), and cisplatin (50 µg/mL) were added and incubated for 1, 3, and 5 h, respectively, except for SDS, which was incubated for 10 min. The cells were then washed with PBS, immersed in a 0.2% trypan blue solution, and observed under an inverted bright-field microscope (Motic AE31E, Moticam 5 Plus, MOTIC) with a 20X objective. Images were recorded and analyzed using Motic images plus 3.0 software.

### 3.8. TUNEL Apoptosis Assay

Apoptosis was evaluated by Terminal Uridine Nick End Labeling (TUNEL) fluorescent staining using the ApopTag^®^ Fluorescein Direct In Situ Apoptosis Detection Kit (S7160, Millipore, CA, USA) following the manufacturer’s instructions. Briefly, MCF-7 cells were seeded on sterile coverslips until 80% confluency was reached.

Cells were preincubated with 20 µM Caspase-3 inhibitor Z-DEVD-FMK (5q-69401U, BD Biosciences, Franklin Lakes, NJ, USA), 20 µM Caspase 8 inhibitor Z-IETD-FMK (51-69381U, BD Biosciences, Franklin Lakes, NJ, USA) for 30 min, and then appropriate wells were incubated with 10 µM Cisplatin, 50 µg/mL AX-2, or TRIS buffer as a negative control for 3 additional hours.

Cells were fixed in 1% paraformaldehyde (PFA) in PBS for 10 min at RT, PFA was drained, and the cells were washed twice with PBS for 5 min. Then, a precooled ethanol/acetic acid 2:1 permeabilization solution was added for 5 min at −20 °C. After incubation, plates were drained and washed twice with PBS for 5 min. Excess liquid was removed, and equilibration buffer was added to the specimen and incubated for at least 10 s at RT.

The liquid was removed by aspirating around the sample, and then Strength TdT enzyme was added. Samples were incubated in a humidified chamber at 37 °C for one hour. The samples were then agitated for 15 s with Working Strength Stop/washed buffer and incubated for 10 min at RT. Excess liquid was removed by aspirating around the sample. Next, 1 µg/mL DAPI staining solution (ab228549, Abcam, Cambridge, UK) was added and incubated for 5 min. Antifade mounting media was added and coverslips were mounted on glass slides with rubber cement at the edges.

Samples were observed by fluorescence microscopy using a confocal inverted microscope Olympus Fluoview FV1000 (Tokyo, Japan). TUNEL and DAPI stain at the nuclei were quantified using ImageJ 1.54p bundled with 64-bit Java 8 (NIH, Bethesda, MD, USA). Graph and statistical analysis were made in GraphPad Prism 10.4.1 (La Jolla, CA, USA).

### 3.9. Nitric Oxide Test

Nitric Oxide (NO) production was evaluated as a metabolite involved in the induction of apoptosis in normal cells, such as fibroblasts, PBMC [75,76], and MCF-7 cells [18]. After 24 h incubation of the fibroblasts and PBMC with the parasporal protein treatments, the culture plate was centrifuged for 10 min at 2000 rpm. In a new plate, 50 μL of the supernatant and 50 μL of Griess reagent (1% Sulfanilamide and N-1-naphthylethylenediamine-bicyclic 0.1%) were added. The plates were then incubated for 15 min at room temperature. Subsequently, the absorbance was read at 570 nm using a TP-Reader plate reader (Thermoplate, Shanghai, China) [77,78]. DMEM (100 µL) was used as the negative control (NC). The data are expressed as a percentage of free NO in the medium with respect to the negative control: with % NO free in the middle = (Absorbance of the sample × 100)/average of the negative control.

### 3.10. Reactive Oxygen Species

Dichlorofluorescein (DCFH-DA) was used to indirectly measure the total rate of reactive oxygen species (ROS) present. ROS are associated with the stress adaptation of cells to hypoxia, nutrient deprivation, and harmful agents such as chemicals, radiation, and microbial peptides [41,62,79]. After 24 h of incubation of the fibroblast and PBMCs with the parasporal treatments, the culture plate was centrifuged for 10 min at 2000 rpm, and 100 μL of supernatant, 130 μL of Tris-HCl (10 mM, pH 7.4), and 20 μL of DCFH-DA (1 mM) were added. DMEM (100 µL) was used as a negative control. The plates were then incubated for 60 min in the dark at room temperature. The reading was performed using a fluorescence meter (SpectraMax^®^ i3x-Molecular Devices, Thermo Fisher Scientific Inc. Waltham, MA, USA) at an emission wavelength of 525 nm and an excitation wavelength of 488 nm [80,81]. The data were expressed as a percentage of the total rate of ROS in the negative control, with % Total rate of ROS = (Absorbance of the sample × 100)/average of the negative control.

### 3.11. PS-AX Characterization Methodology

#### 3.11.1. Scanning Electron Microscopy

The morphology and characteristics of the crystal parasporal surface were observed by Scanning Electron Microscopy using an SEM Phenom Pro Desktop microscope equipped with an EDS detector (Thermo Fisher Scientific, Eindhoven, The Netherlands). The microscope operated at multiple acceleration voltages (5 kV, 10 kV, and 15 kV), reaching a resolution of less than 12 nm. The samples were mounted on aluminum stubs with double-sided sticking carbon tape and subsequently coated with 10 nm gold using sputter-coating technology.

#### 3.11.2. X-Ray Powder Diffraction

The presence of crystal proteins was confirmed by X-ray powder diffraction (XRPD). The medium at the end of the culture was centrifuged at 5500 rpm for 25 min at 4 °C and washed as described before. The pellet was frozen at −20 °C during 36–48 h and was dehydrated by lyophilization (LOBCONCO) at 1.5 Pa during 12–24 h.

The XRPD patterns were collected in air and at ambient temperature using a Bruker D-8 Advance diffractometer (Bruker AXS, Karlsruhe, Germany) with CuK_α1α2_ radiation. A sodium iodide (NaI) scintillation detector and a polymer sample holder were used. The 2θ-range explored was 5° to 50° with 0.05° step size, 10 s counting time, continuous mode, and spinning of 15 rpm, as reported [82].

#### 3.11.3. Protein Molecular Identification

The primers used for AX-2 protein identification are shown in Table 1. The plasmid DNA was obtained after 48 h of bacterial culture using the NZYMiniprep kit (NZYTech, Lisbon, Portugal). A total of 100 ng of plasmidic DNA was added to the reaction mixture containing 0.2 µM of specific primers, 5 µL of PCR reaction buffer, 2.5 mM MgCl_2_, 0.4 µM of each of the dNTPs, and 5 U of NzyTaq II (NZYTech, Lisbon, Portugal). The PCR conditions used were 35 cycles of amplification, 94 °C for 30 s for DNA denaturation, the respective primer Tm for 30 s for alignment of primers with their target sequences, and 72 °C for 30 s of extension. The agarose gel (1.5%) of the PCR product was conducted, and the band was purified using a NzyGelpure kit (NZYTech, Lisbon, Portugal). Band sequencing was performed at the DNA Synthesis and Sequencing Unit of the Biotechnology Institute (UNAM). The data were analyzed using NCBI BLAST Nucleotide (http://blast.ncbi.nlm.nih.gov, BLAST+ 2.17.0).

### 3.12. Statistical Analysis

Data was analyzed using a One-Way ANOVA (Analysis of Variance) with Graphpad Prism software version 5.0. The Dunnet test was applied to compare each treatment with the control, with a statistical significance of *p* < 0.05.

## 4. Conclusions

The *Bacillus thuringiensis* AX isolate generates a hemispherical, irregular, and insoluble parasporal crystal approximately 1 µm in size, with lattice parameters consistent with those of Cry1Ac. These crystals are composed predominantly of four proteins: AX-1 (~60 kDa), AX-2 (~55 kDa), AX-3 (~27 kDa), and AX-4 (~20 kDa). Among them, AX-2 exhibits strong cytotoxicity toward MCF-7 breast cancer cells while showing no toxicity toward normal cells, including erythrocytes, PBMCs, and MRC-5 fibroblasts. As AX-2 is non-hemolytic and selectively cytotoxic to cancer cells, it meets the criteria for classification as a parasporin. Partial sequence analysis suggests that AX-2 may belong to the PS4 family.

Exposure to AX-2 induces pronounced morphological alterations in MCF-7 cells, which become rounded or oval, with dark, condensed structures. The protein promotes cell shrinkage, compaction of intracellular components, DNA fragmentation, and increased membrane permeability. These observations indicate that AX-2 triggers apoptosis while compromising plasma membrane integrity. Importantly, the induction of apoptosis does not appear to involve oxidative stress.

Collectively, these findings support the hypothesis that AX-2 functions as a parasporin capable of inducing apoptosis in MCF-7 breast cancer cells through an extrinsic pathway, as the observed cell death appears to be initiated by extracellular signaling. Nevertheless, further studies are required to validate this proposed mechanism.

## Figures and Tables

**Table 1:** Primers for AX-2 molecular identification.

Primer Sequence (5′—3′)	Annealing (°C)	Gene	Amplicon Size (bp)
* Fw: TACAAGCAGGGCGTCCAG	57.5	PS1	737
* Rv: TCTGCTGGAATTTGCAATGCT	56.3		
* Fw: TGTTGGGACTGTTCAGTACG	54.5	PS2	237
* Rv: GTAGTAGAGAATGAAACTTCTCCACC	54.7		
* Fw: TGGGCGAATACTGACGTCCT	58.3	PS3	1059
* Rv: GCAGTGCTTGTACCCGCTAC	58.5		
^#^ Fw: AGTGGTCTCCAGGCTCATACTGG	61	PS4	640
^#^ Rv: TGATATTCCCGAACCTGCCCT	61		
* Fw: CGGAGACAACAACAACAACAAATG	65.8	PS5	414
* Rv: CCAGCATAACCTGGTAAAGGCG	66.9		
* Fw: TACAAGCGAGTTAGCATC	53.6	PS6	647
* Rv: GATAAAGTTCAACGGTTCCAGC	54.2		

^#^ Designed based on the sequence reported in NCBI GenBank, accession number AB180980. * Designed based on the sequences reported by Espino Vázquez, A. N. (2014) (Characterization of parasporins in native strains of *Bacillus thuringiensis* (Doctoral dissertation, Autonomous University of Nuevo León)) [83].

## Data Availability

The original contributions presented in this study are included in the article and Supplementary Material. Further inquiries can be directed to the corresponding author.

## Supplementary Materials

The following supporting information can be downloaded at: https://www.mdpi.com/article/10.3390/molecules31030475/s1, Figure S1: Sequencing analysis by MAFFT software of ~600 bp nucleotides sequence of AX-2 purified protein obtained by PCR product and parasporin 4 (NCBI accession number WP_029440439).
