# Supplementary material for: AX-2: A Promising Non-Hemolytic Protein of Bacillus thuringiensis with Potent Selective Cytotoxicity Against Breast Cancer Cells
Source: Molecules. 2026 Jan 29;31(3):475. doi: 10.3390/molecules31030475 (PMC12898861; doi:10.3390/molecules31030475)
Supplement: Supplementary file 1 [file molecules-31-00475-s001.zip › molecules-4030769-supplementary.pdf]

AX-2: A promising non-hemolytic protein of *Bacillus thuringiensis* with potent selective cytotoxicity against breast cancer cells

Alain Cruz-Nolasco 1, Miguel Angel Peña-Rico 2,*, Sibel J. Estrada-Escobedo 3, Angel A. Ortela-Gregorio 3, Erick A. Juarez-Arellano 4, Genaro Vázquez-Victorio 5, Angelica S. Martinez-Ramirez 2, Michele Rorato Sagrillo 6, Roberto C. Vianna Santos 7, Luz Camacho 8, Nayeli G. Nieto-Velázquez 9 and A. Karin Navarro-Mtz 2,*

1División de Estudios de Posgrado, Doctorado en Biotecnología, Universidad del Papaloapan, Circuito Central 200, Parque Industrial, Tuxtepec 68301, Oaxaca, Mexico; alandurst13@gmail.com (A.C.-N.)

2Centro de Investigaciones Científicas, Instituto de Biotecnología, Universidad del Papaloapan, Circuito Central 200, Parque Industrial, Tuxtepec 68301, Oaxaca, Mexico; asmartinez@unpa.edu.mx

3División de Estudios de Posgrado, Maestría en Biotecnología, Universidad del Papaloapan, Circuito Central 200, Parque Industrial, Tuxtepec 68301, Oaxaca, Mexico; sibel99926@gmail.com (S.J.E.-E.); anto-nio_ortela_@hotmail.com (A.A.O.-G.)

4Centro de Investigaciones Científicas, Instituto de Química Aplicada, Universidad del Papaloapan, Circuito Central 200, Parque Industrial, Tuxtepec 68301, Oaxaca, Mexico; eajuarez@unpa.edu.mx

5Facultad de Ciencias, Universidad Nacional Autónoma de México, Circuito Exterior S/N, Ciudad Universitaria, Ciudad de Mexico 04510, Mexico; genvazquez@ciencias.unam.mx

6Graduate Program in Nanoscience, Franciscan University, Santa Maria 97010-032, RS, Brazil; sagri-llomr@ufn.edu.br

7Oral Microbiology Laboratory, Universidade Federal de Santa Maria, Santa Maria 97065-060, RS, Brazil; rober-tochrist@gmail.com

8Laboratorio de Nutrición Experimental, Instituto Nacional de Pediatría, Ciudad de Mexico 04530, Mexico; ca-macho.luz@gmail.com

9División de Investigación, Hospital Juárez de México, Ciudad de Mexico 07760, Mexico; goretinieto@gmail.com

*Correspondence: mapena@unpa.edu.mx (M.A.P.-R.); anavarro@unpa.edu.mx (A.K.N.-M.); Tel.: +52-287-875-9240 (ext. 220) (M.A.P.-R. & A.K.N.-M.)

```
CLUSTAL format alignment by MAFFT (v7.511)


PS4Expprot AM-------------RLCVYVSPSYFEPLHIDLGMKKLYLHL------------------
WP_0294404 MAILDLRAVAENYWKKYCASKGYNYIRTDMPDVEISNFNINILDPVVFANPGNTALAFGT
                          : *.  . .*:..   *: :.:: :::

PS4Expprot ------------------QVSNKMT---HRFPSLFQSQSCGSLSIHLLYVQLLHTSLLQC
WP_0294404 TPNRTSRDLLRTLTFNETQTDSQSTTTEHGITAGYSVTARAEASILFATVGIETTMSLEY
                             *...: *   * :.: :.  : .. ** :  * :  *  *:

PS4Expprot MHQQHHTLLPMRLKLHQ----VHLRPLWHAVNMYL-QSG----NALLYLNF---------
WP_0294404 NYTNSKTYTKEVSRSWEDSITITVPPGYETRHTFIVQTGPFNKNVALECDITGLTTCWFS
            : : :*      :  :    : : * :.: : :: *:*    *. *  ::

PS4Expprot ------YAYDQFVLQLELLNYQCTLXLHKXHSXTLVVVFPFSDLYG--------------
WP_0294404 HPQVPGYTFGESDYMSRILDYEGIPNISLIGSNNIARFKGVGNLVGSMALQSYIDLEERP
                 *::.:     .:*:*:    :    * .:. .  ..:* *

PS4Expprot ---------------------------------
WP_0294404 LPGRSGQTRRYQIPVTGRSGIDIPILDPVLSRQ
```

Figure S1. Sequencing analysis by MAFFT software of ~600 bp nucleotides sequence of AX-2 purified protein obtained by PCR product and parasporin 4 (NCBI accession number WP_029440439).
